# A Review of the Sustainable Utilization of Rice Residues for Bioenergy Conversion Using Different Valorization Techniques, Their Challenges, and Techno-Economic Assessment

**DOI:** 10.3390/ijerph19063427

**Published:** 2022-03-14

**Authors:** Sivabalan Kaniapan, Jagadeesh Pasupuleti, Kartikeyan Patma Nesan, Haris Nalakath Abubackar, Hadiza Aminu Umar, Temidayo Lekan Oladosu, Segun R. Bello, Eldon R. Rene

**Affiliations:** 1 Institute of Sustainable Energy, Universiti Tenaga Nasional, Kajang 43000, Malaysia; jagadeesh@uniten.edu.my; 2 Chemical Engineering Department, Universiti Teknologi Petronas, Seri Iskandar 32610, Malaysia; 3 Independent Investigator, 15008 A Coruña, Spain; harisnalakath@gmail.com; 4 Mechanical Engineering Department, Bayero University Kano, Kano PMB 3011, Nigeria; ummihadiza@gmail.com; 5 Mechanical Engineering Department, Universiti Teknologi Petronas, Seri Iskandar 32610, Malaysia; temidayo_18000263@utp.edu.my; 6 Department of Agricultural and Bioenvironmental Engineering Technology, Federal College of Agriculture Ishiagu, Ishiagu 402143, Nigeria; segemi2002@fcaishiagu.edu.ng; 7 Department of Environmental Engineering and Water Technology, IHE Delft Institute for Water Education, P.O. Box 3015, 2601 DA Delft, The Netherlands; e.raj@un-ihe.org

**Keywords:** rice residues, techno-economic evaluation, biomass pre-treatment, energy augmentation, rice residues valorization, renewable energy source, sustainable development, bioenergy and biofuels

## Abstract

The impetus to predicting future biomass consumption focuses on sustainable energy, which concerns the non-renewable nature of fossil fuels and the environmental challenges associated with fossil fuel burning. However, the production of rice residue in the form of rice husk (RH) and rice straw (RS) has brought an array of benefits, including its utilization as biofuel to augment or replace fossil fuel. Rice residue characterization, valorization, and techno-economic analysis require a comprehensive review to maximize its inherent energy conversion potential. Therefore, the focus of this review is on the assessment of rice residue characterization, valorization approaches, pre-treatment limitations, and techno–economic analyses that yield a better biofuel to adapt to current and future energy demand. The pre-treatment methods are also discussed through torrefaction, briquetting, pelletization and hydrothermal carbonization. The review also covers the limitations of rice residue utilization, as well as the phase structure of thermochemical and biochemical processes. The paper concludes that rice residue is a preferable sustainable biomass option for both economic and environmental growth.

## 1. Introduction

Human diets have developed in tandem with per capita income, food production cost, and human population expansion. The world’s food choices vary due to cultural variety, and dietary preferences fluctuate between countries and specific locations, which can also be tied to raw food availability [1]. Likewise, for low-income countries such as Nigeria, Afghanistan, Sudan and others, an increase in gross domestic product GDP (USD 458 billion) [2] is accompanied by food consumption pattern substitutions [3]. Meanwhile, rice is a widely recognised staple food crop, particularly prevalent in Southeast Asia, where it accounted for more than the world consumption average of 54.8 kg/person in the year 2016 [4]. Approximately 90% of the world’s rice is produced in Asia, where 140 million hectares of land are devoted to rice growing [5].

Rice husk (RH) is cylindrical in shape and ranges in size from 4 to 10 mm depending on the variety of paddy harvested. Approximately 0.41–3.96 of the residue-to-product ratio can be produced from 1 kg of harvested paddy in a normal milling process [6]. The major weight breakdown of rice depends on the variety of paddy harvested, which consists of 78% with respect to rice, broken rice and bran, and 22% of husk collected during rice milling [7], respectively. Furthermore, a maximum rice-to-grain ratio yield depends on the harvesting technique, land fertility, light intensity and water supply [8].

The simultaneous increase in both rice production and consumption creates massive amounts of residues, such as straw, husk, bran and stalk. There is an assortment of long-term drawbacks that accompany poor management and improper disposal methods, such as the leaching of soil nutrients and soil organic matter [9], environmental pollution, and human health deterioration. Interestingly, rice residue’s positive outcome through alternative energy transitions has gained wide research interest over the years.

The leftover paddy residues in the form of straw on farmland could have the potential for agricultural restoration as mulch, which retains the farmland’s nitrogen content. However, the nitrogen in rice residue (RR) is not as significant as in mineral fertilizer application [10]. Rice straw (RS) left on the farmland could lead to environmental problems due to methane (CH_4_) emission, which is more hazardous than CO_2_ emission, sharing around 80% of the total green house gas (GHG) emissions from the cultivation stage [11]. The shortcut taken in reducing the RR by openly burning or piling on the rice land is attributed to the emission of harmful gases and huge amounts of particulate matter (PM) dispersion [12,13].

According to Quispe et al. [14], out of the annual 134 million tonnes of produced RH in the world, roughly 90% is disposed of via open burning, or discharged into either the river or the sea. The author has also proven the amount of particulate matter (PM), carbon monoxide (CO), carbon dioxide (CO_2_), ash and sulphur dioxide (SO_2_) emissions, which are approximately 3 kg, 60 kg, 1460 kg, 199 kg and 2 kg, respectively, corresponding to every ton of paddy and wheat straw (WS) burning [15]. The emissions from RR burning will cause severe respiratory-related problems in the long run for those who live in the paddy field’s vicinity. Golshan et al. [16] have analysed that the symptoms of respiratory morbidity illnesses such as asthma, insomnia and cough, and noted that they persisted among people in the suburbs of Isfahan, Iran, due to the smoke from rice-waste-burning activities.

Nevertheless, open burning is the cheapest and easiest method to clear and prepare the farmland in the shortest period for the next cultivation. This has resulted to the loss of significant macronutrients and micronutrients such as nitrogen, phosphorus, and potassium on agricultural land [17], and leads to the loss of valuable potential feedstock to be used in the invention of various materials.

In some rural parts of India, RH is generally used for cooking and heating due to its uniformity, low cost and abundance across the country [18]. Conversely, RR has a low nutritive value as ruminant feed (cattle) since its cellulose and other contents are significantly lower [19] compared to the straws of legume crops and stover. Simultaneously, RR could potentially be a renewable resource, with reserved high economic value, to generate future energy needs. Recently, RS and RH have been used as construction materials, adsorbents of heavy metals [20], and in the production of energy and fuels. Table 1 illustrates the application of RR on various potential platforms, which creates new employment opportunities among farmers.

RR, which consists of RH and RS, has delivered various positive outcomes and challenges towards a sustainable environment. The acquisition of constructive elements involved in the usage of RR could be an asset to the future of environmental conservation if proper usage and analysis are performed. Therefore, this study sets out to conduct a thorough and systematic review of the characteristics of rice production; rice residue utilization in physico-chemical, thermo-chemical and biochemical energy conversions; RR pre-treatment techniques and RR limitations for fuel production; and the techno-economic contribution of RR for energy augmentation and CO_2_ emission attenuation propensity. Finally, the present and the future recommendations for RR utilization are highlighted in the study.

## 2. Physico-Chemical Characteristic of Rice Residues

The physical and chemical properties of any biomass material are critical in establishing the overall qualities of biomass fuel, and in providing a basic idea of whether additional pre-treatments are necessary. Structural characteristics are the basic characteristics of the plant materials, and chemical characteristics are the functional chemical constituents present in the biomass feedstock.

### 2.1. Structural Characteristics

Structural characteristics are the predominant area of study in biomass constituents, and are a precursor to biomass material, associated with the proportion of natural polymers present in the biomass. The major natural polymers found in biomass are hemicellulose, cellulose and lignin. Table 2 shows the polymer compositions of RH and RS with some selected biomass. Lignin is found to be highest in softwoods (25–32%) than hardwoods (18–25%) [35], lowest in straws and bagasse, and almost absent in mosses and algae [36]. The lignin proportion in biomass feedstock contributes to an overall calorific value of the feedstock. A large percentage of lignin in the form of phenylpropanoid, blended with 20% opaline silica in RH, prevents water penetration and fungal decomposition, and leads to disposal difficulty [37]. Additionally, a higher proportion of lignin will contribute to a very low yield of sugar, halting biofuel (bioethanol) production. However, the content of hemicellulose and cellulose in the RH and RS has no direct contribution to or relationship with the higher heating value (HHV) [38,39].

### 2.2. Proximate Analysis of Rice Residues

In general, the majority of RH has the lowest moisture content with the highest range of ash and fixed carbon content compared to the RS, CC (corn cobs), WH (wheat husk) and SB (sugarcane bagasse), as shown in Table 3. The quality of biomass feedstock is indirectly proportional to the moisture content of the material, which significantly reduces the drying costs in preparing the fuel for power generation purposes. The moisture content of biomass residues will contribute to the cost of per unit of energy generated, transportation, pre-treatment (discussed in the upcoming section of this article), the cost of storage, and the adaptation of appropriate conversion technologies. Apart from that, a higher content of fixed carbon is vital to producing better fuel for the combustion process.

In contrast with conventional fossil fuels, the drawbacks of RH and RS are relatively smaller when focusing on volatile matter constituents where CC and SB produce a substantial amount of volatile matter (roughly 69–89% [42]), which could lower the ignition temperature [46]. This lower ignition temperature could be altered by mixing with other higher-ignition-temperature biomasses with specific mixing ratios [58]. On the contrary, higher volatile matter contributes to higher consumption of secondary air and higher pressure for complete combustion to take place [59]. Incomplete combustion will lead to other problems, such as heat loss through the emission of dark smoke, which causes environmental pollution and PM deposition on the boiler tubes [59]. Additionally, volatile content causes biomass to burn faster than usual as about 75% of the volatiles are released and smouldered in the initial pyrolysis stages [38]. Moreover, high ash (incombustible material) contents in RR are not desirable, especially for combustion plant parts in which they can cause defects, such as fouling in a heat exchanger and reducing the heating value of fuel [60].

### 2.3. Ultimate Analysis and HHV of Rice Residues

A higher heating value (HHV) indicates the energy content of a given biomass. The typical HHV of agricultural residues ranges between 15 and 17 MJ/kg. Based on the literature review, the HHV of RH and RS are 14.61–15.44 MJ/kg and 12.10–16.60 MJ/kg, respectively [61,62]. The HHV influences the ash content and extractive content of biomasses. A study of four different types of softwood and hardwood conducted by Robert [63] revealed that the extractive content increases the average HHV by 1.80% and 1.67% compared to extractive-free woods, respectively. Additionally, Demirbas [64] reported that ash content reduces the HHV of any biomass. Although a higher percentage of C is believed to be thermochemically effective and lead to a better combustion process, a higher percentage of sulphur can cause other disadvantages to the boiler system, such as the formation of sulphur oxides (SO_X_). The sulphur content of RH (0.08–0.20%) is significantly lower than that of CC (0.15–0.69%), substantiating the fact that RH is a more desirable feedstock for biomass boiler applications, as shown in Table 4 below.

## 3. Pre-Treatment of Rice Residues

Global research efforts have focused on the impact of pre-treatment from the perspective of technicality, economic viability and environmental sustainability, despite the potential positive attributes of RR. However, there are limitations, such as combustion instability, due to high moisture content, substantial smoke generation when burnt directly, low density and energy values, and hydrophilic behaviour; these have hindered their utilization as direct feedstock for power generation and fuel or chemical production [70,71,72]. To overcome these undesirable properties, further treatment is required that adds value to the physical, mechanical, thermal, and chemical characteristics [73,74] of biomass feedstocks. Biomass pre-treatment from waste to energy conversion, particularly mechanical densification (briquetting and pelletization), as well as thermal processes (torrefaction and hydrothermal carbonization), have gained recognition within the research sphere over a long period of time. Table 5 summarises these pre-treatments’ adaptability to raw biomass compensation to increase the higher end utilization and functionality [75].

### 3.1. Briquetting

Briquetting is a promising technique that has gained wide recognition in recent years because of its superior compressive strength when compared to other methods. Briquetting employs an additional binding agent, such as saw dust, cow dung, starch, asphalt or molasses, to increase its compressibility strength [76,77,78,79]. Due to the low bulk density of RH, its handling and transportation become a challenge. However, such problems are controlled through the production of briquettes, which becomes easy to use in later valorization processes. Even though briquetting has been commonly used as solid biofuel in the power generation field, these briquettes are commonly used to replace firewood or coal for heat generation purposes. Briquettes are regarded to be more efficient and environmentally friendly since they release less PM compared to that released during RH combustion.

Ahiduzzaman and Sadrul Islam [85] found that the implementation of RH briquettes supplements the current wood fuel to prevent deforestation in Bangladesh. Additionally, through the fuel-switching of wood fuel to RH briquette, it has been found that no additional cost is required (cost-benefit), the existing parboiling process has been improved with almost 50% less consumption of RH, and up to 24.14 thousand hectares/year of forest land deforestation have been prevented [85]. Briquettes are better than pellets in various ways, such as the reduction in pressure needed for densification, lower price value, and correlative production location, such as palm mills (production decentralization) [86]. However, RH-based briquettes have a low bulk density and moisture content, which lead to poor mechanical strength and durability [80]. Therefore, additional binder, feedstock mixing ratios, ultimate pressure, temperature setting and shapes are essential to produce a better briquette.

The addition of binder increases moisture content and reduces the combustion efficiency of the fuel. Olugbade and Mohammed [87] discovered the usage of starch binder with mixed rice bran (RB) and palm kernel shell (PKS) produces better compressibility strength; it also burns effectively, is cost-effective and is readily available. The briquette produced at a 3:7 ratio (PKS: RB) with a PKS grain size of 2 mm, and at a 6:1 ratio (PKS and RB) to the binder, had the highest calorific value of 14.25 MJ/kg [87]. Another study by Akolgo et al. [88] found that a mixture of charred sawdust, RH and coconut husk briquettes made from multi-feed biomass gasifier produced a higher calorific value of 24.69 MJ/kg with less CO emission (0.1 g), and consumed less fuel, with a combustion efficiency of 34.7%, in comparison with raw wood charcoal. Both of the above studies demonstrate the potential to prevent the usage of wood logs for cooking purposes, thereby indirectly reducing the exploitation of forests in the long run; however, the drawbacks are still prominent, and might add to the overall cost of the briquettes through the addition of binders.

### 3.2. Pelletization

The rapid development of rice pellet production has faced much advancement over the years in Asian countries, where the production of rice is concentrated [89]. The chemical breakdown of rice pellets has significantly improved combustion performance compared with raw RR. However, a higher production temperature and pressure are needed to compress the RR, to form a better pellet with higher compressibility strength and to prevent the unnecessary addition of a binding agent; this is significant in reducing the final calorific value of the produced pellet.

Most of the raw biomass applications used as solid fuels in a biomass boiler, especially straw, produce a higher amount of ash content; this causes corrosion and clinker, which could lead to the need for scheduled maintenance in the long run. Reducing the amount of ash content in any solid biomass fuel is necessary for the boiler’s fuel application. Yang et al. [90] discovered that leaching with water for 15 weeks, by exposing the RS under outdoor sunlight, contributed to a significant decrement in ash content from 12.3 to 9.6% in the fifth week. Additionally, the durability of the RS pellet is higher than the RH pellet, whereby the durability of both pellets improved with the increase in temperature [90].

Bajo et al. [91] examined the influence of different mixing ratios of RH to ground wood (0:1, 1:4, 2:3, 3:2, 4:1) by mass and different applied pressures, ranging from 80 to 120 MPa. As a result, the moisture content, volatile matter, fixed carbon and HHV of mixed pellets decreased by 54.7%, 17.7%, 4.7% and 19.4%, respectively, as RH mass increased from 0% to 100%. However, 100% RH pellets had the highest ash content (17.8%) compared to other mixed-pellet groups. In addition, a higher applied pressure ranging from 80 to 120 MPa increased the compressive strength by 6.5 to 31.6%, with an increase in the RH mass of mixed pellets [91]. Wang et al. [81] conducted a similar analysis in their study, using blended RS and WS with a mixing ratios of (1:0, 0.5:0.5 and 0:1) at various temperatures (80–120 °C), pressures (20–40 MPa) and particle sizes (0.285–0.685 mm). Based on the experiment, a higher applied temperature resulted in a higher pellet density.

### 3.3. Torrefaction

Torrefaction, a French word for roasting, is a biomass pre-treatment process in which feedstock is subjected to temperatures between 200–400 °C in a nearly oxygen-lean environment with a reaction time of 20 to 60 min [83]. Torrefaction treatment is aimed at reducing the fibrous nature of biomass to improve the biomass properties for energy generation. During torrefaction, the carbonization and volatilization of hemicellulose occur, with nearly 100% of the moisture being eliminated. This is followed by the depolymerization and devolatilization of lignin and cellulose in lignocellulosic biomasses; this results in solid components with significant improvements in their physical and chemical properties, as well as combustion characteristics, compared to the original biomass [92]. This process is capable of producing three major products: (1) dark solids; (2) yellowish acidic-aqueous; and (3) non-condensable gaseous products [72]. The major attributes of torrefied biomass are that it has a high mass yield, high energy density, a hydrophobic nature and durable biodegradation; it is also brittle, easily grindable, has market potential, and has a density similar to coal for use in power boilers and multi-fuel feedstock [93]. The performance of the torrefied products is affected by several key parameters such as biomass type, torrefaction temperature, and residence time [94]. Torrefaction can be applied to all types of wood, grasses and other types of biomasses, resulting in a uniform commodity fuel [95,96].

Chen et al. [97] found that the torrefaction of RH is optimal under a temperature range of 230 to 260 °C for 30 min. Additionally, it has been observed that a significant reduction can be seen in the percentage of volatile matter and moisture of the feedstock as the temperature increases from 260 to 290 °C [97]. Meanwhile, Kwo and Jamari [98] determined that 250 °C is the ideal torrefaction temperature in the conversion of both RH and RS, which yield maximum energies of 92–94% and 93–98%, respectively, with a value-added product such as biofuel. In a later analysis by Chen et al. [99], similar results were found for RH and RS, as the temperature increased where energy yields decreased by 20.84% and 36.99%, respectively. Moreover, the yield of gaseous products of RH and RS increased in the ranges of 0.13–6.44% and 0.58–11.88% with increasing temperature from 250 to 300 °C, owing to the decarboxylation of polymers and dehydrogenations of aromatic structures [99].

A study found that torrefied WS, RS, and gin waste reduced moisture by 70%, 49%, and 48% after an hour of residence time at 260 °C, respectively [100]. As residence time increased from 0 to 60 min, the heating values for WS, RS and gin waste increased gradually to 15.29%, 16.91% and 6.33%. Based on the observation, gin waste lost the least amount of weight (9.67%) due to the highest amount of lignin compared to RS (30.68%) and WS (23.86%) [100]. On the other hand, torrefied RS contributed to a heterogeneous condensation of potassium chloride (KCl) vapour due to higher concentrations of K and Cl in PM, which increased with the rise in temperature during the torrefaction process [101].

Kizuka et al. observed that the mixing of torrefied RS at 280 °C with wood biomass pellets at a ratio of 10% RS to the pellets generated resulted in a lower heating value (LHV) of 16.5 MJ/kg, which is higher than the LHV of wood [102]. Conversely, they also found a higher energy loss at a 280 °C torrefied temperature, whereas a torrefaction temperature of over 220 °C was needed to improve the grindability of RS. Moreover, the LHV decreased as the mixing ratio of torrefied rice straw (TRS) to wood increased. The adaptation of torrefaction pre-treatment prior to the pelletization process will achieve wider application of the final produced pellets and contribute additional regional economic benefits [102]. Another study by Homdoung et al. [103] proved that the average bulk density and durability of torrefied RH pellets were 1112 kgm^−3^ and 90.6%, respectively. In addition, the water resistance (91.9%) and compressibility strength (140 kg/m^3^) were also improved significantly [103].

### 3.4. Hydrothermal Carbonization

Hydrothermal carbonization (HTC) is one of the thermal pre-treatment approaches, also known as wet pyrolysis, commonly used to converts biomass with relatively high moisture content (ideal: 75–90%) [104] into a biochar, a bio-oil or a biogas [105]. Depending on the reaction temperature and pressure, the hydrothermal process can be divided into three sub-classes: HTC, hydrothermal liquefaction (HTL) and hydrothermal gasification (HTG), [105]. Hydrothermal carbonization needs milder reaction conditions such as low temperature (180–280 °C) and saturated pressure (1–8 MPa) [84], which have attracted more research interest in recent years due to lower hydrochar ash yield, higher energy density, high-carbon end products and more smokeless solid fuels than the HTL [106]. In spite of this, HTC is a catalyst-based reaction; for example K_2_C_2_O_4_ [107], NaOH, and ZnCl_2_ [108] or acids, such as H_2_S0_4_ and H_3_PO_4_ [109], are used to displace nonporous by-products. Different process parameters are involved in producing efficient HTC, such as the addition of a potassium compound (catalyst), which is far more economical than any other available catalyst in the market [110].

Xu et al. [110] investigated the distribution of heavy metals in RS when inorganic additives such as K_2_SO_4_, KCl, KOH, and K_2_CO_3_ were added during hydrothermal carbonization. The experiment revealed that adding K_2_SO_4_ significantly increased the HHV, carbon content and energy recovery of the resulting biochar, while leaving the distribution of heavy metals, cadmium (Cd) and copper (Cu) significantly unchanged. In contrast, the addition of KOH resulted in a 27.2% and 18.5% increase in the distribution of Cd and Cu, respectively, as compared to biochar formed from RS without the additive [110].

A study by Nizamuddin et al. [49] optimized the hydrothermal process of RS via microwave-induced hydrothermal carbonization (MIHTC). According to the results, the ideal conditions for hydrochar synthesis were a temperature of 180 °C, a 20-min reaction time, a 1:15 mass-to-volume (*w/v*) biomass-to-water ratio and a 3 mm particle size, yielding 57.9% hydrochar [49]. Moreover, the HHV increased to 17.6 MJ/kg for optimized hydrochar with MIHTC, compared to 12.3 MJ/kg of RS [49]. A decrease in moisture content, oxygen content, and volatile matter was also observed in the hydrochar. However, the ash and nitrogen content in RS hydrochar increased from an initial value of 6.9% and 0.2% to 16.5% and 0.4%, respectively.

Even though the objectives of material pre-treatments are to improvise for the production of volumetric-energy-dense materials with ease of transportation, handling and storage facilities, most of these methods concentrate on the additional cost, equipment-related breakdowns and harmful effects to humans; therefore, they require extensive corrosion-resistant devices or reactors. The application of pre-treatment methods should not only emphasize the efficiency of raw material conversion into value-added products, but should also be focused on reliability, efficiency and ease of use from every aspect. Further research initiatives are required in developing alternative tools or methods in RR pre-treatment at various scales of industrial fuel application; this can help to reduce the amount of fossil fuel usage in the future to make sustainable, reliable and affordable fuel sources.

## 4. Valorization of Rice Residues

**Figure 1:**
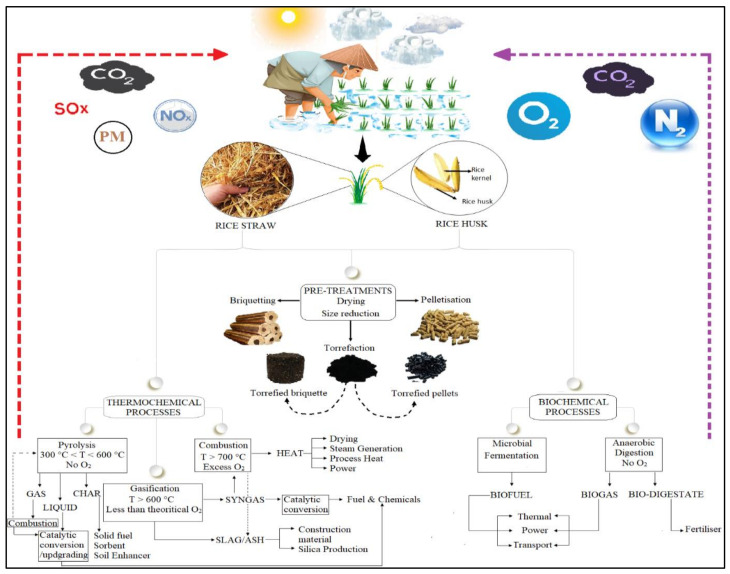
Rice husk and rice straw conversion through thermochemical and biochemical processes. Note: SOX (sulphur oxides), PM (particulate matter), NOX (nitrogen oxides), CO_2_ (carbon diox-ide), O_2_ (oxygen), N_2_ (nitrogen).

The selection of appropriate technology for material conversion is largely dependent on its moisture content and its physical, mechanical, and chemical composition criteria [111]. In particular, biomass materials can have moisture contents as high as 95% by weight and still be suitable for combustion with a supercritical water process. Meanwhile, biomass can be handled using updraft fixed-bed and fluidized-bed gasifiers with moisture contents between 50 and 65% by weight, respectively [112], and moisture contents of 0–20% are considered efficient for fast pyrolysis [113]. In the subsequent section, thermochemical methods such as gasification, pyrolysis and direct combustion, and biochemical processes such as anaerobic digestion (AD) and microbial fermentation, are discussed in detail as prominent technologies used in deriving the chemical energy stored in RR. Direct combustion produces electricity incorporated through conduction, convection and radiation processes, and is a heat-generated (exothermic) system. However, in gasification and pyrolysis, heat is consumed endothermically in an oxygen-lean environment to produce bio-oil, char, chemicals and syngas. Figure 1 illustrates the conversion of RR into various kinds of waste to energy, fuel and chemical transformations.

### 4.1. Direct Combustion

Combustion is an ancient method used for generating heat (exothermic reaction), steam and energy; it is used for drying purposes in agricultural industries and accounts for almost 97% of the world’s bioenergy production [114]. During combustion, the feedstock is confined and exposed to high temperatures between 700 °C and 1350 °C to produce heat under sufficient air supply (usually 110 to 150% of stoichiometric oxygen) [73,115]. Direct combustion furnaces have a combustion chamber where biomass is burnt directly, and are commonly used in agro-industrial processes such as the production of sugar cane, rice and coffee, among others, in regions such as Central and South America [116]. The reaction takes place in surplus air where oxygen is the oxidizing agent. A typical combustion reaction is represented in Equation (1) [117].

(1) $$C_{a}O_{b}H_{c}N_{d}S_{e}+{\mathrm{wO}}_{2}\to \mathrm{Energy}+{\mathrm{vCO}}_{2}+{\mathrm{xH}}_{2}O+{\mathrm{yN}}_{f}O_{g}+{\mathrm{zSO}}_{h}$$

Combustion is applied for industrial activities via the use of stoves, boilers and fluidized combustors. Rice straw thermal conversion research studies are still in the developmental stage, unlike RHs research. Among the different thermochemical processes, direct combustion is well established and mostly used for its economic heat generation from biomass [118]. The heat generated from the biomass (RH) boiler is directly or indirectly used for the parboiling process at processing mills [119]. The rice husk contains approximately 75% organic volatile matter, and the remaining 25% of its weight is converted into ash, which is known as rice husk ash (RHA), during the incineration process [119]. The high ash content, which has a range of 9–22% [120,121,122], and remaining chemical make-up of RH mean that rice husk combustion faces some challenges. For instance, the high amount of silica and sulphur, along with alkali metals and alkaline earth metals, present in RH poses a threat to its combustion, as it leads to the low melting temperature of ash followed by slag generation and fouling [120,122,123]. Moreover, it has been found that the influence of ash on thermal behaviour is directly related to the amount of potassium in the feedstock. Ash containing a high potassium content has low fusibility, while ash containing high calcium or phosphorus has high fusibility [124]. Straw ash generally has a higher potassium content than other fuels, resulting in a slag problem during thermal conversion [124]. Additionally, the high ash content is also obtained during the combustion process, which causes deposition of ash on the heat exchanger, leading to inefficiency of heat transfer, corrosion and shortening of the overall equipment life [123]. Several solutions have been suggested to the problems posed by ash content, such as a reduction in combustion temperature, the pre-treatment of biomass prior to combustion, and the alteration of chemical properties by leaching, among several others [119,123].

Rice husk combustion was conducted in a circulating fluidized bed to determine the effect of bed material diameter, gas velocity and mass fraction on RH mixing [46]. The results revealed a very high process efficiency of about 97%. Maximum mixing was obtained at a sand diameter of 0.3–0.8 mm and a gas velocity of 0.8–2 m/s. The ignition temperature was found to be lower than that of coal (340 °C), while emissions varied from 200–800 ppm for CO, 50–100 ppm for SO_2_ and 150–220 ppm for NO_x_. Similar research was conducted by Armesto et al., where RH was combusted in a bubbling fluidized bed reactor. The effects of a combustion temperature of 840–880 °C and fluidization velocity of 1–1.2 m/s were tested. The results indicated a high efficiency of more than 97%, and the temperature was found to influence the combustion efficiency and CO emission [125].

Another study conducted by Rozainee et al. [126] features the optimized conditions of RH combustion in terms of the fluidization velocity. The authors focused on a minimum fluidized velocity (U_mf_) range of 1.5 U_mf_ to 8 U_mf_. The results showed that the optimum fluidizing velocity was approximately 3.3 U_mf_, which resulted in significant mixing of RH with sand with a high degree of penetration. Madhiyanon et al. determined the combustion characteristics of RH in a short-combustion-chamber fluidized-bed combustor [127]. The combustion characteristics were determined in terms of emission, temperature distribution, process efficiency and heating rate intensity. The results proved the system to be capable of operating without any secondary solid as a bed material, along with significant combustion efficiency and heating rate. A maximum combustion efficiency and heating intensity of 99.8% and 1.54 MW_th_ m^−2^ were obtained. However, the efficiency was dropped when fluidizing velocity and excess air were increased. Additionally, with an increase in excess air, major emissions of CO and NO_x_ increased in the range 50–550 ppm and 230–350 ppm, respectively [127].

Duan et al. [128] investigated RH combustion behaviour and emission in a vortexing fluidized-bed combustor (VFBC), with the effect of excess oxygen ratio (EO) and in-bed stoichiometric oxygen (IBSO) ratio, and with the temperature distributions of fixed-bed (BT) and freeboard (FBT). With a BT of 700 °C and 100% IBSO, a rise in FBT from 849 to 897 °C exhibited a near-linear relationship with an EO of 40 to 70%, whereas CO and NO_X_ emission concentrations decreased, and increased as combustion progressed. Despite this, with a fixed 50% EO and 700 °C BT, the FBT trend dropped from 897 to 847 °C with an increase in IBSO from 70 to 100 °C. The reason for this is that higher concentrations of IBSO caused complete combustion of RH and indirectly reduced secondary air consumption, thereby reducing FBT. Alternatively, a rise in both BT and FBT from 700 to 770 °C and 930 to 962 °C was achieved with a constant 50% EO and an increase in IBSO from 70 to 100%. In both non-fixed and fixed BT, the concentration of CO increased as the IBSO increased, and vice versa for NO_X_. Zain et al. [129] combusted RH to produce ash for use as a supplementary cementitious material. In their study, different combustion parameters such as fire duration (30 and 60 min), air supply duration (60, 90, and 105 min) and chilling duration (1 and 2 days) were investigated in a custom-built furnace, as well as the grinding methods of burned RH ash, using a Los Angeles machine with two rod types and a grinding time range of 15–210 min. Factors such as furnace temperature, ash particle size, silica crystallization phase and chemical content of the produced RHA were investigated. The result showed that duration of combustion, chilling time, and grinding process and duration are vital in securing ash with high standards in terms of quality and texture. Additionally, air ducts in the furnace are vital for proper combustion.

There are limited studies on the large-scale combustion of RR for power generation. Guillemot et al. [130] reported that at least three power plants are producing 24–25 MW in China; these are known to use RS as part of their biomass fuel, and around 466 biomass-power projects have been put into operation at the present time [131]. In the Indian state of Punjab, a total of 11 power plant projects using 100% RS as fuel have been constructed, with an annual RS processing capacity of 876,500 metric tonnes. Among them are Sukhbir Agro Energy Limited in the Ferozepur district with an 18 MW capacity, and Universal Biomass Energy Pvt. Ltd. in Mukatsar district with a 14.5 MW capacity [132]. Furthermore, in Vietnam, a 50 kW RH cogeneration demonstration plant was installed in Long An province in 1999, and the first official RH power plant was built in Can Tho city by the Dinh Hai Cogent Joint Stock Company in 2006, capable of processing 36,908 metric tonnes of RH per year [133,134]. However, problems with feedstock collection, feeding—especially at large-scale operations—and low economic efficiency led to temporary closure due to inadequate resource supply [135]. This experience suggests that small combustion systems similar to the farm-scale paddy flatbed dryer (PFBD) developed by the Rice Engineering and Mechanization Division Philippine Rice Institute, which can be placed near rice fields, may be more feasible [136]. A PFBD is a simple and low-cost system that consists of a drying bin, a blower and a furnace. Rice straw is used as fuel in the furnace, and the heat generated is used to dry the rice paddy [137].

### 4.2. Gasification

RH gasification has been conducted by different scholars to investigate its capability to generate syngas for use as a clean fuel. Gasification is the thermochemical conversion process for carbonaceous biomass in the presence of a controlled medium (air, oxygen or steam) to produce synthesis gas (or syngas) in a gasifier (see Equation (2)) [138].

(2) $$\begin{array}{ll} \left(\mathrm{Biomass}\right)\left(s\right)+ & m\left(O_{2}+3.76N_{2}\right)\left(g\right)+{\mathrm{nH}}_{2}O\left(g\right)\\ & \to x_{1}\mathrm{CO}\left(g\right)+x_{2}{\mathrm{CH}}_{4\;}\left(g\right)+x_{3}{\mathrm{CO}}_{2}\left(g\right)+x_{4}H_{2}\left(g\right)+x_{5}H_{2}O\left(g\right)+3.76{\mathrm{mN}}_{2}\left(g\right) \end{array}$$

Gasification takes place at elevated temperatures of 700–1100 °C [139], with fractions of oxygen supply theoretically required for complete combustion. Other gasifying agents are employed, including air, steam (H_2_O) and carbon dioxide (CO_2_). The resulting gaseous products (CH_4_ and H_2_) are capable of generating heat or steam utilizable in gas turbines to produce electricity. The equivalence ratio (ER) is a critical parameter in air gasification. It is defined as the ratio of oxygen (air) supplied for the stoichiometric need for complete combustion of the fuel which, for gasification, is between 0 and 1. Thus, increasing the O_2_ supply leads to a reduction in the concentrations of H_2_, CO and CH_4_ and a rise in the concentration of CO_2_ in the resulting syngas. Another important factor is the bed temperature, which has an influence on the syngas composition and heating value. While increasing the temperature leads to an increase in syngas yield, it also results in the formation of undesirable gases such as NOx and SOx. To minimize the tar content of the produced syngas, catalytic cracking using calcite, zeolites, dolomite or olivine, or high-temperature cracking, is employed in combination with integrated gas cleaning equipment during the gasification process [140,141].

Korberg et al. [142] and Young [143] provide good reviews of the basic reactions involved in gasification. A study on gasification at 850 °C, and with an air-fuel ratio of 0.579 for RS in a fluidized-bed gasifier, resulted in achieving hot and cold gas efficiencies of 61% and 52%, respectively, and generating syngas (10% H_2_, 18% CO and 4% CH_4_) with an HHV of about 5.1 MJ/Nm^3^ [144]. In their study, to avoid bed agglomeration during RS gasification, a mixture of alumina-silicate and MgO was employed as bed media in the fluidized bed gasifier [109]. The steam gasification studies on RS in the presence of potassium carbonate (K_2_CO_3_) showed improved hydrogen-rich gas production with yields up to 59.8% [145].

Compared to pyrolysis, gasification technology is relatively more advanced, and while there are no commercially installed RR gasification plants like for other biomass materials (e.g., wood chips), downdraft and updraft gasifiers are beginning to be available at commercial scales, while fluidized bed gasification systems are still in the demonstration phase of International Finance Corporation (IFC 2017). According to Ouda et al. [146] the most promising markets for gasification plants are in Asia, followed by Europe, Africa and America. Brandin et al. [147] reported that gasification technology is sufficiently advanced for commercialization, except for some unit operations requiring further development. In 2010, Dimpl [148] reported that worldwide standard gasifier technology appropriate for small-scale applications proved to be unreliable and expensive.

Park et al. [140] investigated the operational characteristics of a bubbling fluidized-bed gasifier system for 20-tons-per-day RH gasification, at an operational bed temperature of 700–850 °C and an ER of 0.20–0.35. According to the results, the ideal operating parameters for RH gasification were 0.20 ER and a bed temperature of 800 °C. The producer gas had an LHV of 1373.18 kcal/Nm^3^ and a cold gas efficiency of 70.75%. The producer gas was cleaned to remove tar at an efficiency of 98%, which enabled the cleaned producer gas to be used for a 400 kW_e_ gas engine. Gajera et al. [149] investigated the kinetics of co-gasification of RH and petroleum coke, using a thermogravimetric analyser at three different heating rates (10, 20, and 30 °C/min). The co-gasification process improved the reactivity of petroleum coke, as the activation energy of 126 kJ/mol for gasification of petroleum coke alone was lowered to 86 kJ/mol for the co-gasification of a 3:1 mixture of RH and petroleum coke.

Non-catalytic high-temperature gasification was applied to reduce the tar and suspended particulate matter of the produced gas. Makwana et al. [141] carried out RH gasification at varied high temperatures ranging from 720 to 855 °C in a pilot scale fluidized bed gasifier, to identify the optimal setting for better syngas properties suitable for thermal and power operations. The optimal gasifier temperature was determined to be 790 °C, which resulted in a syngas with low tar content, an LHV of 3.2 MJ/Nm^3^, high carbon conversion efficiency (91.6%), high thermal efficiency (75%) and a high gas yield (2.7 m^3^/kg). Susastriawan et al. [150] compared the gasification performance of RH, sawdust (SD) and the mixture of these two biomasses (1:1) by varying the ER between 0.15, 0.20 and 0.25 in a small-scale fixed-bed downdraft gasifier. The results proved that the gasifier was compatible with all three feedstocks and at the optimum ER of 0.2 for RH, the producer gas had an LHV of 3.13 MJ/Nm^3^, and the cold gas efficiency of the gasifier was 72.73% for RH.

A simulation study of RH gasification in an entrained gasifier was conducted by Gao et al. [151] where sensitivity analysis was carried out to determine the influence of process parameters such as the gasification temperature (800–1100 °C), average particle diameter (220–350 μm), ER (0.2–0.4) and mass ratio of CO_2_ to biomass (0–0.8) on the gas composition, gas production and cold gas efficiency. The response surface methodology was employed to optimize producer gas yield and composition, and the cold gas efficiency of the gasifier. At the optimal operating conditions determined by the response surface methodology, producer gas containing a CO concentration of 25.15%, a producer gas yield of 1.96 Nm^3^/kg and a cold gas efficiency of 65.34% were obtained. Hoque et al. [152] gasified RH, coconut shell (CS) and SD in a fixed-bed downdraft gasifier to compare the syngas generation by different biomasses. A varying temperature of 650–900 °C was used for the gasification, with a feed rate of 3–5 kg/h, for all the three biomasses. The results indicated that higher syngas components CH_4_ and H_2_ were generated by CS rather than SD and RH. Subsequently, CS resulted to lower emissions than RH, even though emission by the RH was lower compared to that released by the fossil fuel. The composition of producer gas generated from the RH gasification was 18.48% CO, 10.45% CO_2_, 0.166% CH_4_, and 14.0% H_2_, with an LHV of 933.6 kcal/Nm^3^ and cold gas efficiency greater than 60%. The authors ascertained that all the three biomasses can generate clean fuel for power generation.

### 4.3. Pyrolysis

Converting biodegradable material into liquid fuel allows modern applications in power generation, liquid fuel production and chemical production, from biomass. Pyrolysis of biomass is among the prominent technologies for clean fuel production; these are mainly biochar (solid), bio-oil (liquid) and non-condensable gas (syngas), as shown (see Equation (3)) [153].

(3) $$\mathrm{Biomass}⇄H_{2}+\mathrm{CO}+CH_{4}+H_{2}O\left(g\right)+\mathrm{Tar}+\mathrm{Char}$$

Unlike other endothermic processes such as gasification, pyrolysis decomposes organic matter without the use of any oxidizing agent in an inert environment, by feeding on either argon or nitrogen gas. The process begins with moisture evaporation (dehydration) at a low temperature of about 100 °C, followed by biomass cracking and devolatilization as the temperature reaches around 300 °C [154]. The outcome of the process usually depends on the pyrolysis temperature, heating rate and residence time. A low temperature with a longer residence time (slow pyrolysis) is suitable when the required by-product is biochar, while a short residence time with a high temperature (fast pyrolysis) favours the release of bio-oil and pyrolysis gases such as CO_2_, CO, H_2_.

Weldekidan et al. [154] conducted a pyrolysis study on RH using sufficient heat generated by concentrated solar radiation, which allowed for full recovery of the pyrolysis product. RH was pyrolyzed at different temperatures and the pyrolysis gas yield obtained was 14 wt.% at 500 °C, increasing to a maximum of 25.48 wt.% at 800 °C. The maximum biochar yield was found to be 43 wt.% at 500 °C, while the bio-oil yield varied within a range of 20.6–43.13 wt.%, with the highest yield obtained at 700 °C. The pyrolysis gas product had an HHV of more than 197 MJ/kg [154]. Zhou et al. [155] investigated the effect of the most widely utilized catalyst, NiO/γ-Al_2_O_3_, on RH pyrolysis in order to characterize the dehydration process. The results indicated that dehydration occurred at a temperature of 250–350 °C whereby about 5.92% of the dry raw material was reduced, which accounts for the moisture loss under the non-catalytic pyrolysis process. Moreover, the catalyst used (γ-Al_2_O_3_) had an influence on the dehydration process, as the water yield increased to 15.36%; moreover, with a 5–20% NiO loading on γ-Al_2_O_3_, the water yield decreased from 13.16% to 12.14% at a pyrolysis temperature of 350 °C. Fu et al. [75] investigated the production of activated biochar using RH through one- and two-step KOH catalytic pyrolysis. In the one-step process, RH was blended with KOH (1:1, mass ratio) and pyrolyzed at 750 °C in a fixed-bed reactor; in the two-step process, RH was first pyrolyzed at 450 °C, and the resulting biochar was blended with KOH (1:1) and pyrolyzed at 750 °C. By comparing the results obtained under this condition, it was concluded that two-step catalytic pyrolysis yielded (19.4%) more activated biochar than one-step pyrolysis (2.5% yield). Furthermore, it was observed that increasing the amount of KOH lowered the biochar yield in the two-step pyrolysis performed at different mass blend ratios of 1:1, 1:1, and 3:1 (KOH: biochar). For the adsorption of phenol from water, activated biochar synthesized in the two-step process, with a 3:1 mass ratio of KOH to biochar, showed the maximum adsorption capacity of 201 mg/g due to its high surface area (S_BET_ = 2138 m^2^/g), and sufficient micro-pores. Biswas et al. [156] conducted a comparative pyrolysis study in a fixed-bed reactor to determine the influence of temperature on biochar and bio-oil yields by slow pyrolysis of CC, RH, WS, and RS at temperatures ranging from 300 to 450 °C. For CC, WS, RS, and RH, the maximum bio-oil yields were obtained at 450, 400, 400, and 450 °C, yielding 47.3, 36.7, 28.4, and 38.1 wt.%, respectively. Furthermore, in their respective optimum temperature, the biochar obtained were 24.0, 34.4, 33.5 and 35.0, respectively. For all the biomasses examined, the highest amount of organic carbon was obtained in RH biochar at 300 °C, reaching 58.74 wt%, while the highest organic conversion was observed in RH at 400 °C, reaching 56.62 wt%. The pre-treatment of biomass prior to pyrolysis using treatments that aid in demineralization and deoxygenation is one way to improve the quality of the pyrolysis products, which was proposed in [137]. Zhang et al. [157] evaluated the effects of pre-treatments on the kinetics of pyrolysis and characteristics of the products using RH feedstock that was washed with acids and then torrefied. The activation energy of the RH sample increased as a result of the washing and torrefaction pre-treatments, reaching 147.9 kJ mol^−1^ at a torrefaction temperature of 210 °C—due to the removal of alkaline earth metals, and crosslinking and carbonization caused by torrefaction—compared to 104.6 kJ mol^−1^ for raw RH. The ash of the biochar using combined pre-treated or only acid-washed RH contained an increase in SiO_2_ content, which can be used for the preparation of silica products.

Co-pyrolysis of biomass with other materials has been used to increase the yield and quality of the pyrolysis product, which would otherwise have a high oxygenated content and a low hydrocarbon fraction when pyrolyzed alone. Lin et al. [158] co-pyrolyzed oily sludge with RH at 600 °C in a fixed-bed reactor and observed that increasing the RH in the blend decreased the yield of bio-oil, while increasing the yield of pyrolysis gas. This was attributed to the presence of ash and alkali metals in the biomass, which act as catalysts; these promoted secondary cracking of hydrocarbons in the bio-oil and resulted in their further decomposition to a low-molecular-weight pyrolysis gas, which subsequently resulted in an improved bio-oil quality. The co-pyrolysis with an increase in oily sludge resulted in a decrease in saturates and aromatics content in the bio-oil. Additionally, the interaction between oily sludge and RH enhanced the hydrogen yield in the gas product and improved the distribution of sulphur in both bio-oil and pyrolysis gas. Vieira et al. [159] optimized operating conditions such as temperature, heating rate, residence time and RH mass to obtain the best condition for slow pyrolysis-based biochar production in a fixed-bed reactor using Taguchi’s method. Based on process efficiency and energy consumption, the optimal conditions for biochar production were found to be 500 g of RH, a temperature of 400 °C, a heating rate of 20 °C/min and a residence time of 3600 sec. However, the biochar with the best HHV of 23.41 MJ/kg was obtained at different settings (RH mass = 125 g, temperature = 500 °C, heating rate = 10 °C/min and residence time t = 5400 sec). Wang et al. [160] co-pyrolyzed RH with sewage sludge (SS) to investigate the characteristics of a gas product and thermal degradation behaviour during the process, by employing thermogravimetric Fourier-transform infrared spectrometry–mass spectrometry. The results indicated that the addition of RH led to an increase in the pyrolytic index from 2.19 × 10^−7^ to 10.14 × 10 ^−7^%^2^ min^−2^ °C^−3^, and a CO_2_ production of SS with reductions in hydrogen, methane and C_2_H_2_ gases. The interaction dominated biogas production while promoting the carbonization and aromatization of the resulting biochar when the 30% RH was added. It was concluded that an increased dosing of RH may lead to pore development and a significant increase in specific surface area, and that co-pyrolysis of RH and SS absorbs gaseous hydrocarbons and yields high-capacity absorption biochar [161]. Table 6 compares the process conditions for pyrolysis, gasification and combustion [162,163].

### 4.4. Anaerobic Digestion

Anaerobic digestion (AD) consists of four distinct stages (Table 7): hydrolysis, acidogenesis, acetogenesis, and methanogenesis. During these stages, microorganisms break down complex organic matter found in wastewater, municipal solid waste, and other sources to produce end products such as methane-rich biogas and digestate. Additionally, biogas contains other gases such as CO_2_, H_2_, and trace amounts of NH_3_ and H_2_S. The solid digestate may then be processed further to use as fertilizers.

Various factors asso ciated with the effectiveness of AD are correlated with the strength of bacterial activity, such as C/N ratio, total volatile fatty acid (VFA), pH, organic loading rate (OLR), temperature and hydraulic retention time (HRT). Realizing the inevitable complexity of microorganism growing conditions, multi-stage digester configuration has been suggested to ease the microbial conversion process [167].

Temperature is one of the crucial factors in predicting the end product of the AD system. However, a high temperature (thermophilic condition: 50 to 60 °C) could provide multiple benefits, for example, high metabolic rate, faster stabilizing rate, high specific bacterial growth and higher biogas production [168]. Nevertheless, there are certain disadvantages too, such as high mortality rates of certain bacteria that grow well in a low-temperature range (mesophilic condition: 25 to 40 °C).

The ligneous nature of RR with the highest lignin content and recalcitrant nature, causing its AD to slow down with conventional digestion methods, has resulted in the adaptation of various pre-treatments such as physical, chemical, biological, mechanical or thermal breakdown of the lignin content. Additionally, pre-treated RR, incorporated with cellulose and hemicellulose structure alteration, exhibits fast hydrolysis to produce biogas within a short time frame. A study conducted by Hsu et al. [169] used diluted sulphuric acid (H_2_SO_4_) as a pre-treatment for fast hydrolysis and found that the maximum pre-treatment temperature range was from 160 and 180 °C to yield a maximum xylose percentage of 89% in RS. Additionally, an increase in pore volume due to the release of acid-soluble lignin has been observed, resulting in an efficiency of 70% for the enzymatic hydrolysis. However, as the temperature increased to 190 °C, the yield of xylose significantly dropped to 55–65% [169].

In addition to various pre-treatments of biomass materials used in AD, bioaugmentation can be implemented via the introduction of microbes with enhanced cellulolytic activity, which can increase the digestibility and biodegradability of lignocellulosic materials and yield more biogas [170]. Shetty et al. [171] evaluated the digestion of RS under routine bioaugmentation of a cellulolytic bio-enzyme consisting predominantly of *O. joyonii*. The average yield of methane was obtained at a much shorter HRT of 15 days by digesting untreated RS with *O. joyonii* (315 L/kg VS/day), compared with ambient-temperature-NaOH pre-treated RS (300 L/kg VS/day), high-temperature-NaOH pre-treated RS (246 L/kg VS/day) and untreated RS (194 L/kg VS/day) [171]. However, NaOH has been reported to facilitate the hydrolysis of the lignocellulosic component, especially the lignin contents. However, microbial growth and methanogenesis were inhibited during anaerobic digestion of alkali pre-treated RS due to a high accumulation of VFA and lower pH values [171].

Rice residues in the form of RS have the highest C/N ratio (44.0–74.0) [172] in comparison with water hyacinth (12–42) [173], cow manure (24.3) [174] and pig manure (10.95) [175]. In the AD process, an optimal C/N ratio in the range of (20–30/1) is needed for bacterial activities. Irregularity or a higher range of C/N ratio than the afore-mentioned causes a lower decomposition rate due to ammonia and VFA build-up, inhibiting the bacterial decomposition and eventually causing process failure. Bhaskar Jha et al. [176] introduced co-digestion of RS with de-oiled rice bran (DORB) in a 2:3 ratio in a mesophilic AD environment to bring down the C/N ratio to a favourable range. Moreover, the results suggested that maximum volumetric methane yields of 9216 litres and 14,385 litres are possible during a digestion period of 90 days, with total solid (TS) concentrations of 7.5% (32.31 kg of VS) and 5% (21.55 kg of VS), respectively [176]. In another study by Saadia et al. [177], RS was co-digested with WS and SB in a batch fermentation test using a glass bottle of 300 mL and a working volume of 210 mL. On the fourth day of the AD process, they found that the co-digestion of both WS and RS in substrate/inoculum ratios of both 1.5 and 2.5 produce a higher yield of methane with 41% and 17%, respectively [177]. However, co-digestion of SB and RS produced a poor yield of methane due to VFA accumulation in the substrate, which halted the overall methanogenesis process [177].

A review conducted by Fu et al. [178] revealed that dry AD technology (DADT) plays a key role in converting agricultural straws into useful biogas in China. From the review, DADT was found to be a feasible method to realize the efficient utilization of low-value agricultural waste, which improves the environment, reduces overall cost, improves economic sustainability, and creates a sustainable energy community network. The idea of DADT was further substantiated by Syafrudin et al. [179] through batch AD experiments of both liquid and solid-state. The experiment was conducted at room temperature with a series of TSs such as 5, 7, 9, 19, 21 and 23%, with sodium hydroxide pre-treated RH and raw RH [180]. Based on the experiment, pre-treated RH yielded higher biogas with a TS of 5% in both solid and liquid states [179].

### 4.5. Microbial Fermentation

Industrial biotechnology is an expanding field that focuses on the use of microorganisms to produce energy and chemicals using renewable resources. Microbial fermentation—also known as acetone, butanol and ethanol (ABE) fermentation—is one of the biotechnology fields that utilizes enzymes to break down and transform large organic materials through metabolic pathways into final fermented products (solvents). Unlike the AD system, microbial fermentation begins with anaerobic glycolysis (the conversion of glucose to pyruvate) which results in the production of two adenosine triphosphates (ATP) and two pyruvate molecules (C_3_H_4_O_3_) for every glucose molecule. The C_3_H_4_O_3_ resulting from the oxidation of NAD^+^ to NADH does not feed into a citric acid cycle; instead, it undergoes decarboxylation to produce acetaldehyde (C_2_H_4_O), depending on the types of fermentation, which are: (1) alcohol fermentation or (2) lactic acid fermentation. Finally, NADH reacts as a catalyst to reduce C_2_H_4_O into ethanol. The final reduction process enables the regeneration of NAD+, and the conversion process is continuous as long as glucose is present. Equations (4)–(6) depict the conversion of glucose into solventogenesis, and Equations (7) and (8) show the fermentation of glucose into acidogenesis with the use of different enzymes, denoted as follows: (i) Pyruvate ferredoxin oxidoreductase; (ii) Acetyl CoA acetyl transferase; (iii) β-hydroxyl CoA decarboxylase; (iv) Butyraldehyde dehydrogenase; (v) Butanol dehydrogenase; (vi) Acetaldehyde dehydrogenase; (vii) NADPH dependent ethanol dehydrogenase; (viii) Acetoacetyl CoA acetate or Butyrate CoA transferase; (ix) Acetoacetate decarboxylase; (x) Phosphotransacetylase; (xi) Acetate kinase; (xii) Phosphate butyryl transferase; and (xiii) Butyrate kinase [180].

(4) $$C_{6}H_{12}O_{6}\to C_{3}H_{4}O_{3}\overset{i}{\to }C_{23}H_{38}N_{7}O_{17}P_{3}S_{\;}\overset{\mathrm{ii}}{\to }C_{25}H_{40}N_{7}O_{18}P_{3}S\overset{\mathrm{iii}}{\to }C_{25}H_{42}N_{7}O_{17}P_{3}S\overset{\mathrm{iv}}{\to }C_{4}H_{8}O\overset{\left(v\right)}{\to }C_{4}H_{10}O\;\;\;\;\;\;\;$$

(5) $$C_{6}H_{12}O_{6}\to C_{3}H_{4}O_{3\;}\overset{\left(i\right)}{\to }C_{23}H_{38}N_{7}O_{17}P_{3}S_{\;}\overset{\left(\mathrm{vi}\right)}{\to }C_{2}H_{4}O\overset{\left(\mathrm{vii}\right)}{\to }C_{2}H_{5}\mathrm{OH}\;$$

(6) $$C_{6}H_{12}O_{6}\to C_{3}H_{4}O_{3\;}\overset{\left(i\right)}{\to }C_{23}H_{38}N_{7}O_{17}P_{3}S_{\;}\overset{\left(\mathrm{ii}\right)}{\to }C_{25}H_{40}N_{7}O_{18}P_{3}S_{\;}\overset{\left(\mathrm{viii}\right)}{\to }C_{4}H_{5}O_{3}\overset{\left(\mathrm{ix}\right)}{\to }C_{3}H_{6}O\;\;$$

(7) $$C_{6}H_{12}O_{6}\to C_{3}H_{4}O_{3\;}\overset{\left(i\right)}{\to }C_{23}H_{38}N_{7}O_{17}P_{3}S_{\;}\overset{\left(x\right)}{\to }C_{2}H_{5}O_{5}P\overset{\left(\mathrm{xi}\right)}{\to }C_{2}H_{3}O_{2}\;$$

(8) $$C_{6}H_{12}O_{6}\to C_{3}H_{4}O_{3}\overset{\left(i\right)}{\to }C_{23}H_{38}N_{7}O_{17}P_{3}S_{\;}\overset{\left(\mathrm{ii}\right)}{\to }C_{25}H_{40}N_{7}O_{18}P_{3}S\overset{\left(\mathrm{iii}\right)}{\to }C_{25}H_{42}N_{7}O_{17}P_{3}S_{\;}\overset{\left(\mathrm{xii}\right)}{\to }C_{4}H_{9}O_{5}P\overset{\left(\mathrm{xiii}\right)}{\to }C_{4}H_{8}O_{2}\;\;$$

Just like other biochemical processes, microbial fermentation (MF) is also influenced by temperature, pH, nutrient shortage, fermentation period, media composition, product inhibition and others, with the production of extracellular metabolites due to microbial metabolic activities [181]. These criteria have been a bottleneck in the production and application of fermented products in various industries such as food industries (cheese and yogurt) [182,183], baking industries (yeast), alcoholic beverages (beer, wine) [184] and alcoholic fuels (bio-butanol) [185]. Although acetone, ethanol and butanol are produced throughout the course of microbial fermentation, butanol has been given the highest priority by many researchers in the past and even the present. Bio-butanol has many qualities that make it popular among researchers, such as lower tailpipe emissions compared with gasoline [186], higher calorific value (32.5 MJ/kg) than ethanol (26.8 MJ/kg) [187], it is non-corrosive, and it can be mixed with gasoline with a maximum percentage of 30% [188].

The initial production of bio-butanol (1st generation) was concentrated on the usage of food crops, and was later directed to non-edible materials such as corn stover [189], wheat straw [190], switch grass [191] and other waste biomasses. Rice residues are an economical and abundantly available option to be used as a non-food material in MF. However, the recalcitrant of lignocellulosic materials with the additional enzymes needed for hydrolysis is attributed to the inefficient and non-economical microbial process [185]. Therefore, numerous applications of pre-treatments have been adopted, such as steam explosion [190], radio-frequency [191], organosolv [192] and microwave irradiation [193], to ease MF.

Moradi et al. [194] observed that phosphoric acid (H_3_PO_4_)-pre-treated RS (PAPRS) recovered a higher solid percentage of 54.3% *w/w* than NaOH pre-treated RS (APRS) (40.1% *w/w*), and none in non-pre-treated RS (RRS). After 72 h of hydrolysis, the glucose yields for every 100 g of APRS, PAPRS and RRS were 35.4 g, 40.8 g and 10.2 g, respectively. During the fermentation process, the cumulative ABE produced from 1 kg of PARPS and APRS were 63 g and 64.1 g, while for RRS it was significantly lower a about 9 g [194]. Though PAPRS yielded a higher solid percentage, butanol yields were higher in APRS (45.2 g) than in PARPS for every kilogram of RS. Later, Valles et al. [195] optimized RS MF using different NaOH concentrations of 0 and 2% *w/v* through different process parameters, such as temperature variations (121 or 134 °C), duration of fermentation, and solid loading percentage (5 and 10% *w/v*). Pre-treated RS with the lowest concentration of NaOH required an extended time of 40 min to produce approximately 42% cellulose (ran 3–4 times), while a higher concentration of NaOH was needed to produce similar output with almost double the running times [195]. Additionally, only a 0.2% *w/v* NaOH concentration, temperature of 121 °C, 40 min and 10% *w/v* of solid loading were required to produce the highest solid recovery of 85.7%. With those parameters, however, very little butanol was produced (0.3 gL^−1^), which could be increased 17-fold by reducing the solid loading to 5% w/v and heating the process to 134 °C [195].

To maximize the production of butanol in RS, Zhu et al. [193] evaluated the effects of different levels of microwave irradiation (300, 500 and 700 W) in combination with NaOH pre-treatment, or using NaOH alone for enzymatic hydrolysis using *Trichoderma reesei*. Compared with the NaOH alone pre-treated RS, the 700 W microwave irradiation for 30 min produced the lowest percentage of moisture (4.8 ± 0.3), lignin (4.9 ± 0.3) and hemicellulose (10.2 ± 0.8) with the highest percentage of cellulose (69.2 ± 0.3). Nevertheless, there was no significant difference in final weight loss; this remained unclear between pre-treated RS with NaOH irradiated with a microwave power of 300, 500, and 700 W, although increasing the treatment time resulted in similar weight loss [193].

The efficiency of ABE production via organosolv pre-treated RS with *Clostridium acetobutylicum bacterium* hydrolysis using different temperatures (150 and 180 °C) was evaluated by Amiri, Karimi and Zilouei [192]. Taking a 30 min retention time, a significant amount of acid-soluble (1.7 ± 0.1 and 1.5 ± 0.1%) and acid-insoluble (12.4 ± 0.2 and 9.9 ± 0.2%) lignin was removed at 150 and 180 °C relative to untreated RS, respectively. In contrast, with the lowest pre-treated temperature and retention time of 150 °C and 30 min, the percentage of solid recovery was highest (78.3 ± 0.9%) [192]. The highest amount of butanol (80.3 g) was obtained with a kilogram of pre-treated RS treated at 180°C for 30 min at a loading rate of 5%, while the highest amounts of acetone (24.6 g) and ethanol (33.5 g) were obtained as a result of treating the RS for 60 min at 150°C and 30 min at 180 °C, respectively, at an 8% solid loading rate [192].

In conclusion, combustion can be used to obtain heat or electricity from RR. Similarly, RR has also been proven to have potential as fuel for syngas generation through gasification; biochar and oil through pyrolysis; and ABE through MF. However, the energy efficiency of these conversion processes is poor due to the lower calorific value, and lower density in comparison with other biomass materials or conventional fuel (coal). Owing to the low moisture nature of RR, it is more suitable for thermochemical processes compared to biochemical processes. As such, when used for the latter process, it may require more pre-treatment in terms of chemical reagents and enzymes. This may lead to more pre-treatment costs, rendering the process less economical, especially if it is to be used at industrial scales. For both thermochemical and biochemical processes, pre-treatment cost is a crucial factor to consider; therefore, optimization techniques have to be implemented in order to achieve quality yield at minimum cost.

## 5. Techno-Economic Consideration of Rice Residues for Energy Augmentation

Despite the promising potential of crop residues in augmenting the global energy resources reported in the literature, it is noteworthy that the evaluation of economic viability associated with crop-residue beneficiation technicalities and logistics is crucial for the waste-to-wealth initiative [196,197]. Rice residue is not an exception, owing to being one of the most commonly grown crops in the world. Different approaches and models have been utilized to evaluate the techno-economic capacity of agro-residues; the preliminary economic assessment involves the estimation of the Levelized Cost of Energy (LCOE) for energy production from the available crop residues [198]. Suzan et al. [198], utilized this method to assess CO_2_ emissions and energy balance based on life cycle assessment (LCA) and savings in the tonne of oil equivalent (toe) of primary energy estimation, and comparison with conventional energy sources, in Egypt. The average LCOE for the biomass power plant was evaluated as (¢/kWh) 6.77; for fossil fuel power plants (¢/kWh) as 8.5 PV; for power plants as (¢/kWh) 8.3; for wind power plants as (¢/kWh) 5.5; and for CSP plants as (¢/kWh) 16.0 [199]. More notably, the LCOE of RR was also reported in the agro-energy opportunity of Egypt and evaluated as 6.33 ¢/kWh, which is very competitive compared with the LCOE of other resources [199]. The inference from the review found RR to be a major contributor to the country’s biomass waste utilization for energy production. Therefore, this may be employed by other developing countries to meet their energy demands.

In any case, Net Present Value (NPV) and Internal Rate of Return (IRR) are also used in place of LCOE as financial indicators, often for the evaluation of an energy project and to develop insight into the line of the project’s profitability [200,201,202]. These three tools were integrated by Rahman and Paatero [203] to evaluate the economic potential of agro-residues in five South Asian countries (Bangladesh, India, Nepal, Pakistan and Sri Lanka). The forecasted annual electricity potentials from the residues in these afore-mentioned countries were valued at an average of around 780 kWh per household. The study optimized the yield by considering anaerobic digestion (AD) in lieu of direct combustion processes. LCOE was EUR 0.040 kWh^−1^ for a 1 kW plant, which is attractive compared with other renewable energy projects within the capacities of 1–5 kW threshold. Regarding NPE and IRR, for a 1 kW electric plant with an electricity selling price between 0.03 and EUR 0.14 kWh^−1^, NPV was reported to be zero at EUR 0.040 kWh^−1^ electricity selling price, and the IRR estimation was 10%. Howeverr, the electricity production prices from other 1–100 kW scale renewable energy productions—such as micro-hydro, solar PV biomass gasifier and small wind—varied over the range of EUR 0.06–0.45 kWh^−1^ [204], and 1 kW of diesel plant varied over EUR 0.30–0.45 kWh^−1^.

Muhammad et al. [205], by the same token, compiled electric power potential from annual residue production from different agricultural produce, considering Pakistan as a case study. Rice straw and husk alone could yield up to 1723 and 256 MW of electric power, respectively. These results corroborate the findings of other literature, and categorizes RR among low-cost agro-product residues. Although maize stalk price is lower than RS, the corresponding LCOE of maize could not match up with that of RS. Therefore, RS becomes more economical when considering the energy potential possibilities [206,207].

Naqvi et al. [208] investigated efficient off-grid power generation using locally available agricultural waste resources in South Asia to meet SAARS’s (South Asian Association for Regional cooperation) initiatives. The total current value of the investigated gasification system varies between $35.98 and $111.83 million, which is highly dependent on the RH costs and the capacity factor. For instance, a higher capacity factor will result in higher net present value. The LCOE of the RH-based gasification system varies between 0.097 USD/kWh to 0.12 USD/kWh, which is largely dependent on the RH cost and capacity factor. Thus, the Levelized Cost of Electricity shows that the LCOE of the studied system is higher than the cost of captive power plants using furnace oil or natural gas [208]. In Australia, Ella et al. [209] examined the RR techno-economic potential of a hybrid concentrated-solar-biomass plant. The rice straw resources of 27,000–255,000 kWh/m^2^/year at up to 16 substations were found to enhance energy efficiency by 34%, with an electricity price range of AUD 120–350/MWh. Brazil’s LCOE biomass energy from residues was also examined for technological requirements in the 2018–2050 timeframe, but the distinctive contribution of each crop residue was never highlighted [210].

A bioethanol plant (BEP) currently takes the largest share in the global biomass-to-liquid biofuel production, as shown in Table 8. The BEP yield is the main parameter to assess the techno-economic feasibility of any residue investigated for this purpose. The techno-economic capability of any designed BEP involves the calculation of yields, operational expenditure, and capital cost as a whole [210]. The importance of BEP is not limited to the cost vantage that it presents to global energy production; it also paves the way for carbon footprint amelioration. Towards the end of the last decade, about 138 billion litres of biofuel were produced in the world, while the America continent accounts for about 87% of the world’s biofuel production. The continental share is given as follows: Africa produced about 0.06; America, 103; Asia, 13.4; Europe, 21.2; and Oceania, 0.20 billion litres (IEA, 2019) [211].

Zhou et al. [212] estimated bioethanol potential from cellulosic ethanol yield from RR in India to be about 0.38 g ethanol per gram of dry biomass, which is the highest in comparison to WS and SB, counterparts with about 0.34 and 0.36 g ethanol per gram of dry biomass, respectively.

### Carbon Footprint Recovery via Rice Residue Conversions to Energy 

In a benchmark that forecasted ethanol production potential from RH in India from 2020 to 2030, the potential from RR using dilute acid and steam-explosion pre-treatment methods, for the production of second-generation bioethanol and surplus renewable electricity generation, was estimated to be 10,547 and 11,165 million L, and 5295 and 6928 GWh, respectively; meanwhile, corresponding greenhouse gas emission reductions were assessed to be 11,954 and 14,375 kt CO_2_eq., respectively [213].

## 6. Conclusions

RR is a natural biomass resource that is sustainable and beneficial to the future of environmental conservation. The quality and characteristics of RR varies according to physical and external factors which have opened new platforms for scientific research. This review explained the utilization of RR and its benefits for a sustainable energy concession. Hence, RR has high susceptibility to thermochemical processes compared to biochemical processes, and a promising feedstock to yield biofuels compared to its counterparts. Even though RR is one of the favourable feedstocks in today’s bioenergy and biofuels market, there are limitations in knowledge gaps in biochemical and thermochemical perspectives that require input. Such knowledge gaps include the artificial intelligence technology in RR energy conservation, the enhancement of the atomic behaviour of RR elemental compositions, and fuel ratios of thermochemical processes; these have cleared the path for future research interests. Future research interest may also include life cycle assessment (LCA) to standardize environmental impact associated with bioenergy, biochemical or biofuel production. Thus, RR is a contemporary biomass option that can be utilized as a biomass fuel substitute for a sustainable future, which is a win–win solution for both the environment and global economic growth.

## Figures and Tables

**Table 1:** Rice residue utilization in various agronomy and material industries.

Agronomy			
Usage	Industry	Year	References
Fertilizer	Agricultural	2017	[21]
Bio-compost (mushroom cultivation)	Agricultural	2014	[22]
**Material**			
Aluminium alloy/clay composite	Construction	2021	[23]
Supercapacitor	Electronic	2021	[24]
RH-based nano-silica catalyst	Acid reforming	2021	[25]
Cement-based composite	Construction	2021	[26]
Tableware (biodegradable cutlery)	Hotels, restaurants, etc.	2019	[27]
Thermal Insulation	Power plants	2019	[28]
Filaments for fused-deposition modelling	3D Construction	2019	[29]
Building blocks/bricks	Construction	2016	[30]
**Fuels/Energy/Treatment**			
Treatment	Wastewater	2021	[31]
Bioethanol	Transportation/power generation	2018	[32]
Biogas	Cooking/power generation	2018	[33]
Energy feedstock	Power generation	2011	[34]

RH: rice husk.

**Table 2:** Natural polymer constituents of selected biomass.

Biomass Type	Cellulose (wt.%)	Hemicellulose (wt.%)	Lignin (wt.%)	Silica (SiO) (wt.%)	Ref.
RH ^a^	25–35	18–21	26–31	15–17	[40]
RS ^a^	36.40	20.40	14.30	6.20	[41]
CC ^a^	45.80	39.40	11.30	1.13	[42]
WH ^b^	42.58	18.54	11.21	NA	[43]
SB ^b^	39.75	38.03	22.01	NA	[44]

Note: NA (not available), RH (rice husk), RS (rice straw), CC (corn cobs), WH (wheat husk), SB (sugarcane bagasse); ^a^ as received; ^b^ dry ash free.

**Table 3:** Proximate analysis of rice residues, corn cob, wheat husk and sugarcane bagasse.

Proximate Analysis (wt.%)					
Biomass Type	MC	VM	AC	FC	Ref.
RH ^b^	4.07–9.50	51.98–71.47	16.30–17.36	3.11–25.10	[45,46,47]
RS ^b^	8.53–13.06	66.75–70.20	6.90–9.22	10.97–14.57	[48,49,50]
CC ^a^	7.14–11.02	69.31–87.76	1.05–5.07	11.19–14.60	[42,51,52]
WH ^a^	4.40–8.45	65.59–69.19	4.99–12.11	12.72–20.97	[43,53,54]
SB ^a^	8.37–10.3	75.72–88.48	1.60–2.20	9.41–16.30	[55,56,57]

Note: MC (moisture content), VM (volatile matter), AC (ash content) FC (fixed carbon); ^a^ dry; ^b^ as received.

**Table 4:** Ultimate analysis of rice husk, rice straw, corn cob, wheat husk and sugarcane.

Ultimate Analysis (DRY basis wt.%)							
Biomass Type	C	H	O	N	S	HHV (MJ/kg)	Ref.
RH	33.14–41.78	5.14–5.50	36.31–37.20	0.30–0.55	0.08–0.20	14.61–15.44	[61,65,66,67]
RS	37.10–39.65	4.88–5.20	35.80–44.30	0.50–0.92	0.10–0.12	12.10–16.60	[62,68,69]
CC	41.07–43.81	6.49–6.54	46.47–50.41	0.25–0.77	0.15–0.69	16.13–16.46	[42,51,52]
WH	47.14–48.50	5.50–5.59	39.90–46.03	0.30–0.37	0.06–0.10	18.90–19.22	[43,68]
SB	41.45–48.81	5.51–6.20	43.10–50.37	0.20–0.51	0.02–0.10	15.96–19.19	[55,57,67]

RH: rice husk, RS: rice straw CC: corn cob, WH: wheat husk, SB: sugarcane bagasse, C: carbon, H: hydrogen, O: oxygen, N: nitrogen, S: sulphur, HHV: high heating value.

**Table 5:** Overview of RR mechanical and thermal conversion processes and products.

Parameters						
Pre-Treatment Technique	Temperature (°C)	Reaction Time (min)	By-Products	Advantages	Disadvantages	Ref.
I	30–700	3–10	CO, CO_2_, H_2_O and solid fuel	Higher compressibility strength	Requires additional binding agent	[75,76,77,78,79,80]
II	80–120	-	CO_2_, water and other by-products	Sensitive for moisture absorption, swell and breakage	Does not require additional binding agent	[81,82]
III	200–400	20–60	Gaseous, aqueous chemicals and solid fuel (char)	Higher energy content, lower moisture content and hydrophobic	Torrefied fuel does not guarantee less corrosion on boiler tubes	[72,83]
IV	180–280	>20	Gases, (aqueous) liquids and solids (hydrochar)	Milder reaction temperature and pressure (autogenous), does not require drying processes, and high solid yield	Corrosion, coke and tar formation, and the process needs high capital investment	[84]

Note: I (briquetting), II (pelletization), III (torrefaction) and IV (hydrothermal carbonization).

**Table 6:** Overview of thermochemical processes, process conditions and products.

Parameters	Pyrolysis	Gasification	Combustion
Process Conditions			
Temperature, (°C)	300–600	>600	>700
Reaction time	1 s (fast pyrolysis), days (slow pyrolysis)	Several seconds to minutes	-
Equivalent ratio (ER)	0	0 < ER < 1	1
**Products**			
Gaseous	CO, CH_4_, C_X_H_Y_, CO_2_, H_2_O, oils, N- and S-containing compounds	CO, H_2_, CO_2_, H_2_O, CH_4_, C_X_H_Y_, tars, NH_y_, NOx, H_2_S, COS	CO_2_, H_2_O, CO, C_X_H_Y_, NO_X_, SO_X_
Solid	C, (N, S), ash	Ash, (N, S)	Fly ash and bottom ash
Liquid	Bio-oil/liquid (tar)	-	-

**Table 7:** Stages in AD system.

Stages of AD				Ref.
Hydrolysis				
Substrate	Microbes	End Product	Specification	
Cellulose, starch, xylan, etc. $(C_{6}H_{10}O_{5})n+{\mathrm{nH}}_{2}O$	I	Simple sugar/monomers $C_{6}H_{12}O_{6}$	Exo-enzymes inhibit the environmental fluctuations and toxins in the feedstocks. Work well in pH (6–8). Slow process. Rate limiting.	[164,165]
**Acidogenesis**				
$C_{6}H_{12}O_{6}$	II	$2{\mathrm{CH}}_{2}{\mathrm{CH}}_{2}\mathrm{OH}+2{\mathrm{CO}}_{2}$	Presence of acid-forming bacteria. Strong and fast growth. Work well in pH (4–8). Inefficient below pH < 4.	[164,165]
$C_{6}H_{12}O_{6}+2H_{2}$		$2{\mathrm{CH}}_{3}{\mathrm{CH}}_{2}{\mathrm{COO}}^{-}+2H^{+}+2H_{2}O$		
$C_{6}H_{12}O_{6}$		$2{\mathrm{CH}}_{3}{\left({\mathrm{CH}}_{2}\right)}_{2}{\mathrm{COO}}^{-}+H^{+}+2{\mathrm{CO}}_{2}+2H_{2}$		
**Acetogenesis**				
${\mathrm{CH}}_{3}{\mathrm{CH}}_{2}{\mathrm{COO}}^{-}+2H_{2}O$	III	${\mathrm{CH}}_{3}{\mathrm{COO}}^{-}+{\mathrm{CO}}_{2}+3H_{2}$	The growth kinetic of acetogenesis is lower than that of acidogenesis. Strict anaerobes which become weaker in acid environment. Work well in pH (6.5–6.2).	[164,165]
${\mathrm{CH}}_{3}{\left({\mathrm{CH}}_{2}\right)}_{2}{\mathrm{COO}}^{-}+2H_{2}O$		$2{\mathrm{CH}}_{3}{\mathrm{COO}}^{-}+H^{+}+2H_{2}$		
${\mathrm{CH}}_{3}{\mathrm{CH}}_{2}\mathrm{OH}+H_{2}O$		${\mathrm{CH}}_{3}{\mathrm{COO}}^{-}+H^{+}+2H_{2}$		
**Methanogenesis**				
${\mathrm{CH}}_{3}{\mathrm{COO}}^{-}+3H_{2}O$	IV	${\mathrm{CH}}_{4}+{\mathrm{HCO}}_{3}^{-}$	Methanogens should be maintained at a stable condition with pH (6.5–7.5). Hydrogenotrophic methanogenesis process produces higher energy than that of aceticlastic methonegenesis.	[164,165,166]
${\mathrm{HCO}}_{3}^{-}+H^{+}$		${\mathrm{CH}}_{4}+3H_{2}O$		
$4{\mathrm{CH}}_{3}\mathrm{OH}$		${\mathrm{CO}}_{2}+2H_{2}O$		
$4H_{3}{\mathrm{COO}}^{-}+4H_{2}+{\mathrm{CO}}_{2}$		${\mathrm{CH}}_{4}+{\;\mathrm{CO}}_{2}+2{\mathrm{HCO}}_{3}^{-}$		

Note: I (*Clostridium* sp., *Acetivibrio cellulolyticus*, *Staphylococcus* sp.); II (*Eubacterium* sp., *Eschericia coli*); III (*Syntrophobacter wolinii, Syntrophomonas wolfei, Smithella propionica*); IV (*Methanothrix soehngenii, Methanobacterium bryantii, Methanobacterium formicicum*).

**Table 8:** Global biomass-to-liquid biofuel production.

Year	In Billion Litres, (Bl)			
	Total	Bioethanol	Biodiesel	Other Biofuels
2000	18.0	13.2	26.7	8.09
2005	38.4	26.7	3.66	8.09
2010	106	66.5	19.9	19.7
2015	128	79.4	30.0	19.0
2016	134	82.7	33.9	17.3
2017	138	85.1	36.1	16.4

Sources taken from (IEA, 2019).

## Data Availability

Data sharing is not applicable. No new data were created or analysed in this study.

## Abbreviations

ABE	Acetone, Butanol and Ethanol
AC	Ash Content
AD	Anaerobic Digestion
APRS	NaOH Pre-treated Rice Straw
ATP	Adenosine Triphosphates
AU	Australia
BEP	Bioethanol Plant
BT	Temperature Distributions of Fixed Bed
C_3_H_4_O_3_	Pyruvate Molecules
CC	Corn Cobs
Char	Gaseous, Aqueous Chemicals and Solid Fuel
CH_4_	Methane
CS	Coconut Shells
CSP	Concentrating Solar Power
CXHY,	Hydrocarbons
DADT	Dry AD Technology
DORB	De-oiled Rice Bran
EO	Excess Oxygen Ratio
ER	Equivalence Ratio
FBT	Freeboard
FC	Fixed Carbon
GDP	Gross Domestic Product
GHG	Greenhouse Gas
GWh	Gigawatt Hours
HHV	Higher Heating Value
HRT	Hydraulic Retention Time
HTC	Hydrothermal Carbonization
HTG	Hydrothermal Gasification
HTL	Hydrothermal Liquefaction
IBSO	In-bed Stoichiometric Oxygen
IEA	International Energy Agency
IFC	International Finance Corporation
IRR	Internal Rate of Return
LCA	Life Cycle Assessment
LCOE	Levelized Cost of Energy
LHV	Lower Heating Value
MC	Moisture Content
MF	Microbial Fermentation
MIHTC	Microwave-Induced Hydrothermal Carbonization
NA	Not Available
NAD+	Nicotinamide Adenine Dinucleotide
NADH	Nicotinamide Adenine Dinucleotide (NAD) + Hydrogen (H)
NADPH	Nicotinamide Adenine Dinucleotide Phosphate
NPE	Nonylphenol Ethoxylate
NPV	Net Present Value
OLR	Organic Loading Rate
PAPRS	Phosphoric Acid Pre-treated Rice Straw
PFBD	Paddy Flatbed Dryer
PKS	Palm Kernel Shell
PM	Particulate Matter
PV	Photovoltaic
RB	Rice Bran
RH	Rice Husk
RHA	Rice Husk Ash
RR	Rice Residues
RRS	Non-Pre-Treated Rice Straw
RS	Rice Straw
SAARS	South Asian Association for Regional Cooperation
SB	Sugarcane Bagasse
SBET	Specific Surface Area
SD	Sawdust
SS	Sewage Sludge
Tar	Bio-oil/liquid
TRS	Torrefied Rice Straw
TS	Total Solid
VFA	Volatile Fatty Acid
VFBC	Vortexing Fluidized-bed Combustor
VM	Volatile Matter
VS	Volatile Solids
WH	Wheat Husk
WS	Wheat Straw
